# Nd-oxide-based nanoparticle-embedded transparent and photoluminescent composite films

**DOI:** 10.1039/d5ra01023c

**Published:** 2025-04-22

**Authors:** Tatsuhiro Matsumae, Hidetoshi Miyazaki

**Affiliations:** a Graduate School of Natural Science Technology, Shimane University 1060, Nishikawatsu Matsue Shimane 690-8504 Japan miya@riko.shimane-u.ac.jp

## Abstract

In this study, we fabricated Nd-based PL composites by embedding Nd compounds in a transparent resin and investigated the relationship between the size of Nd-based particles and PL properties. Nd(iii)-based photoluminescent composite films were fabricated using neodymium nitrate hexahydrate and transparent urethane resin. The resulting Nd-based composite film was pale blue in color. The composite films exhibited near-infrared PL properties with a peak at 1.06 μm (^4^F_3/2_ → ^4^I_11/2_) by irradiation with UV-visible-near-infrared light using a halogen lamp. In the case of the composite film, further irradiation with UV light (using a Hg lamp) for 2 h or less resulted in the film showing strong PL at 1.06 μm under halogen lamp irradiation. Upon UV irradiation for more than 2 h, the film turned brown owing to the reaction of urethane with nitric acid derived from the Nd raw material, and the PL intensity became weaker. The Nd_2_O_3_ nanoparticles in the composite film were measured using transmission electron microscopy, and the particle size ranged from 15.0–35.1 nm. The PL intensity of the composite films was strongest when the size of the Nd-based particles was approximately 20 nm.

## Abstract 2

The resulting Nd based composite films showed PL properties at a wavelength of 1.06 microns, with maximum emission at a particle size of 20 nm for Nd oxide particles in the film.

## Graphical abstract

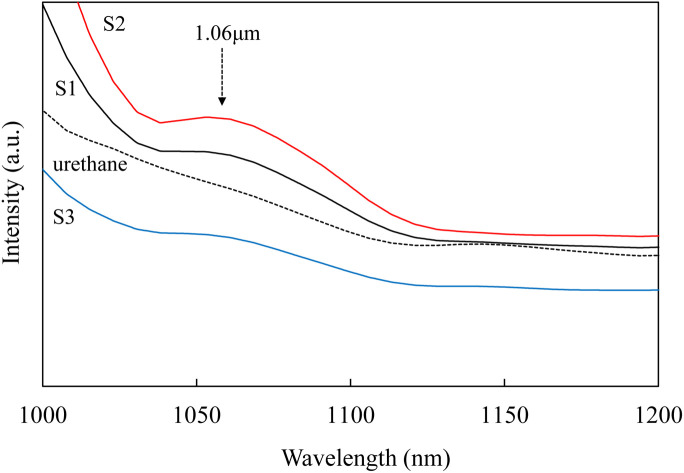

## 1. Introduction

Rare-earth element-doped materials exhibit luminescence attributed to 4f electronic transitions and have therefore attracted considerable attention as PL materials for fiber lasers, solid-state lasers, displays, wavelength-calibrated filters, and optical devices.^1–6^ Such neodymium ion-doped PL materials exhibit fluorescence in the near-infrared region of 1.06 μm (^4^F_3/2_ → ^4^I_11/2_) based on the 4f–4f transition upon irradiation with excitation light and have been applied as various PL and laser materials.^1–3,6^ As a practical material doped with Nd^3+^ ions, Nd: YAG(Y_3_Al_5_O_12_) lasers emit not only strong near-infrared light but also green light at a second harmonic wavelength of 532 nm.^6–9^

Previously, we synthesized composites of Sm_2_O_3_, Pr_2_O_3_, and Dy_2_O_3_ nanoparticles embedded in translucent urethane resin and reported their PL properties.^10–12^ The use of urethane resin as the matrix phase has several advantages, such as high transmittance in the visible to near-infrared region, thus high transparency of the excitation and emission light; preparation not requiring high-temperature equipment such as an electric furnace; recovery of rare earth elements by burning the resin; and low environmental loads because of their fabrication at low temperatures. Using the previously reported method to fabricate composite films,^10–12^ we expected to achieve Nd-based composite films with near-infrared PL properties.

Because high transparency is required for Nd-doped fluorescent and laser materials, they have been studied and applied by synthesizing glass or single-crystal media. These methods require high temperatures and considerable skill, and there are problems such as permeability due to crystallization and phase separation in the glass. However, the proposed method can be performed at room temperature, and permeability can be prevented by degassing, making it simpler than conventional methods. Furthermore, our research has indicated that high transparency can be achieved, and it is assumed that the optical functionality is equivalent to that of samples synthesized *via* other methods. In the present study, we fabricated Nd-based nanoparticle-urethane composite films using neodymium nitrate, which is easily soluble in water and methanol, and photocured urethane resin. In our previous investigations,^10–12^ the prepared composite films were irradiated with UV light to grow particles in the films and improve PL properties. Similarly, in this study, the resulting Nd composite films were irradiated with UV light, and the growth of Nd-based particles in the films and their PL properties were evaluated.

## 2. Experimental procedure

Nd(NO_3_)_3_·6H_2_O (Yttrium Corporation of Japan) was dissolved in methanol to achieve a concentration of 2.0 M and stirred at room temperature for 1 day. The precursor solution (1.0 mL) and 1.0 mL of ethanol were mixed into 3.0 g (3.3 cm^3^) of urethane photosensitive resin (Asahi Photo Microware Co., Ltd) dropwise and stirred for 10 min to homogeneously dissolve the solution in the resin. The mixed slurry was then placed in a desiccator and degassed at 10 kPa. The degassed slurry was molded into 4.2 cm × 2.4 cm × 0.10 cm by placing it between glass plates. The formed slurry was UV-irradiated using a high-pressure mercury lamp (1 kW) for 5 min to cure the resin and obtain Nd-based composite films. The cured composite films were removed from the glass plates and then irradiated with UV light using the same high-pressure mercury vapor lamp for an additional 0–10 h. The films irradiated for 0 h are designated as S1, 2 h as S2, and 10 h as S3.

The transmission spectra of the composite films were recorded using a UV-visible spectrophotometer (UV-1900i, Shimadzu Corporation, Japan). In the near-infrared region, the PL properties of the films were measured using a near-infrared spectrometer (RB4524-050-NIRC3, OtO Photonics INC, Taiwan) with a wavelength resolution (FWHM) of 5.0 nm under irradiation from a halogen lamp light source (150RSVL, Kenko TECHNO LIGHT, Japan). The microstructures of the composite films were observed by transmission electron microscopy (TEM; EM-002B, Topcon, Japan). To perform TEM observations, the composite films were ground to a powder, and the powder sample was subsequently placed onto a copper grid. The crystal structures of the resulting powders were investigated through X-ray diffraction (XRD) conducted using Cu Kα radiation (MiniFlex600; Rigaku Corp., Japan). The Eu-based particles in the composites were characterized using IR spectroscopy (FTIR 4600, Jasco, Japan). We evaluated the transmittance and PL properties of the resulting composites multiple (at least three) times, and samples that exceeded 10% of the mean value were excluded from our evaluation. We used this sample and its transmittance, PL properties, and microstructures, which exhibited the closest mean values for the resulting composite films.

## 3. Results and discussion

**Fig. 1:**
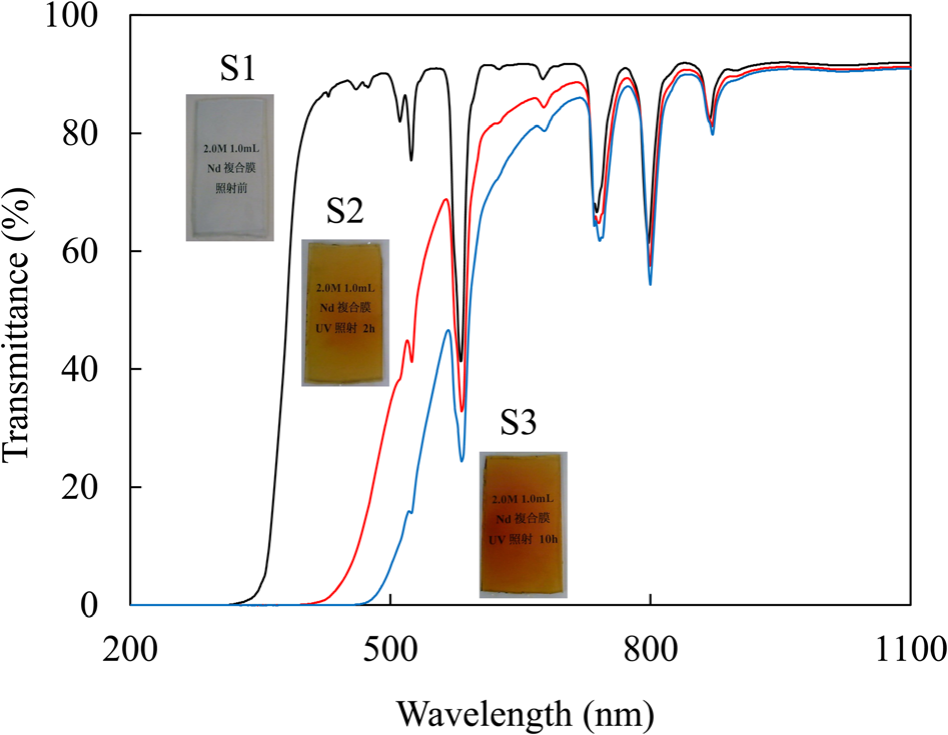
Transmission spectra and photographs of the composite films.

Fig. 1 presents an overview of the photographs and transmission spectra of the as-prepared (S1) and composite films after UV irradiation for 2 h (S2) and 10 h (S3). The as-prepared films are light blue and transparent, and the color of the composite films turns brown with increasing UV irradiation time. This result is attributed to the photoreduction of NO_3_^−^ ion in the film by UV irradiation and azodization between nitrate ions and urethane resin in the matrix.^13^ The composite films show the absorption peaks of Nd-originated at 429 (^4^I_9/2_ → ^2^P_1/2_), 460 (^4^I_9/2_ → ^4^G_11/2_), 471 (^4^I_9/2_ → ^2^G_9/2_), 511 (^4^I_9/2_ → ^4^G_9/2_), 524 (^4^I_9/2_ → ^4^G_7/2_), 581 (^4^I_9/2_ → ^4^G_5/2_), 625 (^4^I_9/2_ → ^2^H_11/2_), 676 (^4^I_9/2_ → ^4^F_9/2_), 738 (^4^I_9/2_ → ^4^S_3/2_), 798 (^4^I_9/2_ → ^4^H_9/2_) and 868 nm (^4^I_9/2_ → ^4^F_3/2_).^1,2,6^ Bouras *et al.* reported Nd luminescence from SnO_2_:Nd thin films, where photon energy of approximately 500–800 nm trapped in SnO_2_ excited Nd^3+^ ions from the ground state to the ^4^G_5/2_ and ^2^F_7/2_ states.^2^ Kassab *et al.* synthesized Nd^3+^-doped PbO-GeO_2_ glass and reported that the effective excitation wavelength of Nd^3+^ at 1.06 μm is the level at 805 nm (^4^I_9/2_ → ^4^F_5/2_).^3^ In this study, we observed an absorption peak corresponding to this state at 798 nm, suggesting that near-infrared (approximately 1.06 μm) luminescence can be obtained by irradiating light at this wavelength, which is similar to the previously reported results. We evaluated the PL properties of the composite films by excitation using a halogen lamp (150 W/relative irradiance >75%: 600–800 nm, maximum wavelength: 700 nm), which had strong visible to near-infrared (964 nm) emission as the excitation light.

**Fig. 2:**
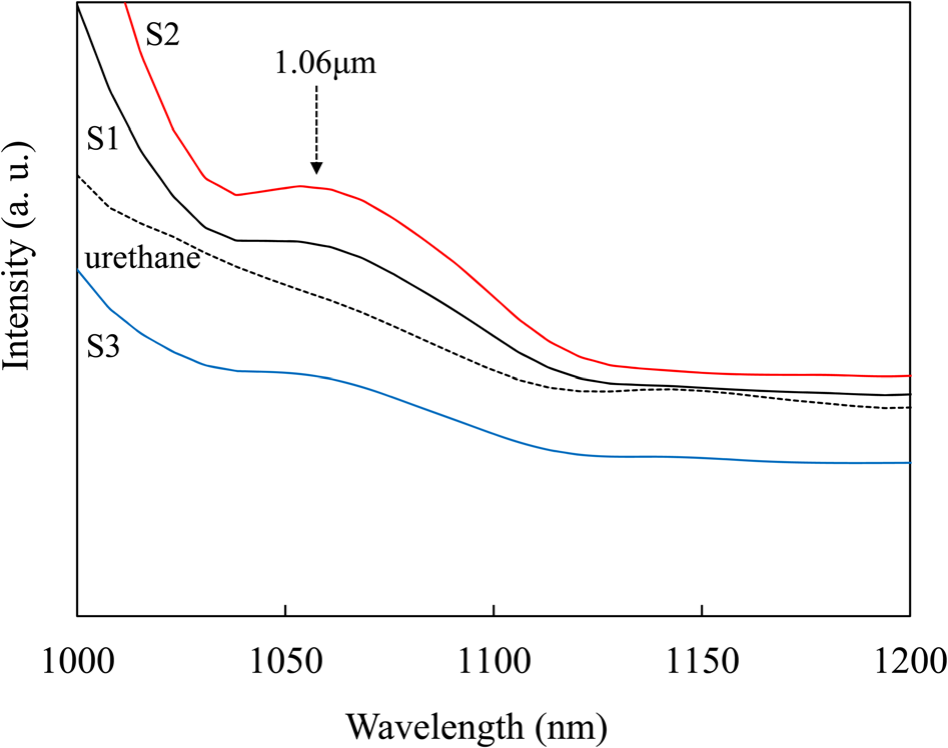
PL spectra of the urethane resin and composite films.

Fig. 2 shows the PL spectra of the urethane resin and the resulting composite film (excited by the aforementioned halogen lamp). The urethane film does not exhibit a PL peak at approximately 1.06 μm. The Nd-urethane composite films show a PL peak at 1.06 μm (^4^F_3/2_ → ^4^I_11/2_) derived from the Nd^3+^ emission peak. The PL at the wavelength of 1.06 μm was almost consistent with the emission in Nd-doped single crystals or glass materials in the previous reports.^6–9^ The highest PL intensity is observed for the composite with UV irradiation for 2 h. Although the maximum PL intensity is observed despite the coloring (caused by urethane degradation by UV irradiation), we discuss these results later using IR absorption and TEM measurements. When the UV irradiation time exceeds 2 h, the luminescence intensity significantly decreases. It is assumed that excessive UV irradiation causes the degradation of urethane and the discoloration is due to NO_3_^−^, resulting in a decrease in fluorescence intensity because it is difficult for the excitation light to pass through the composite films. Therefore, additional UV irradiation time of less than 2 h was considered suitable for the composite films. Urethane, which is a matrix, also exhibits broad emission in the visible to near-infrared region. Because the wavelength region of this fluorescence corresponds to the absorption peak of Nd^3+^, it is assumed that the fluorescence of urethane may promote the fluorescence of Nd^3+^ (sensitizing effect).

When the composite film was further irradiated with UV light for 2 h, the PL intensity of the film at the wavelength of 1.06 μm was the strongest. To clarify these results, the particle size and chemical bonds of the particles in the composite films were investigated by TEM and IR spectroscopy, respectively.

**Fig. 3:**
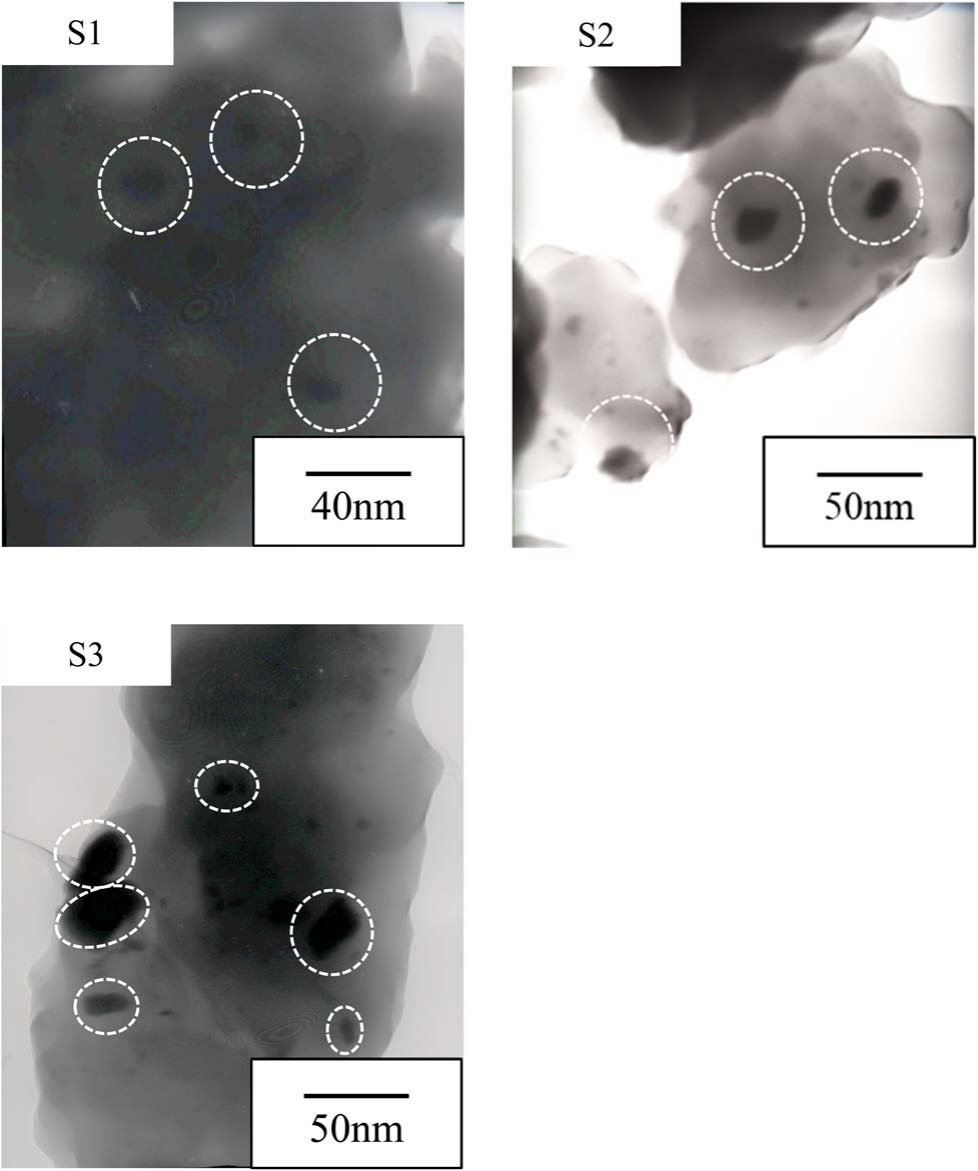
TEM images of the Nd-based composite films.

**Fig. 4:**
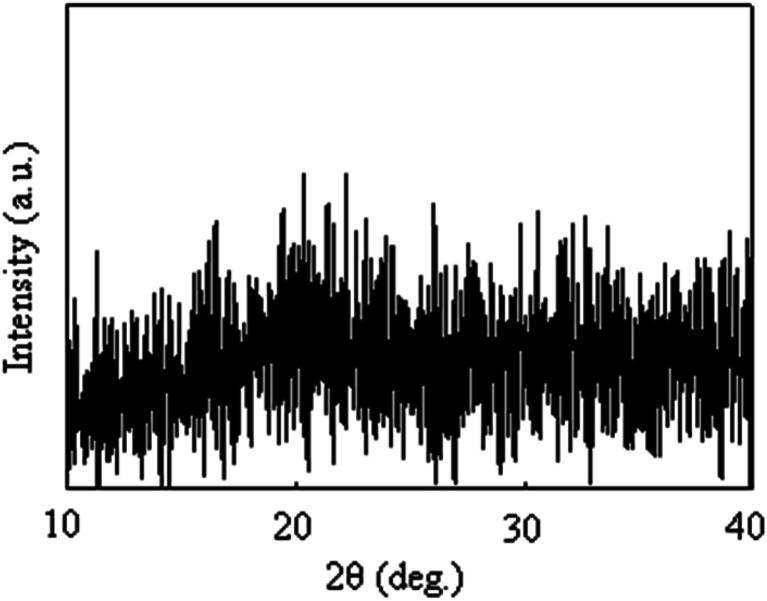
A representative XRD pattern for the Nd-based composite film (S2).

Fig. 3 shows the TEM bright-field images of the resulting composite films. Spherical particles were observed for all the composite films, and the mean particle sizes of the Nd-based compounds in the composite films are S1:15.0 nm, S2:21.6 nm, and S3:26.2 nm. We evaluated the crystallographic structure using electron diffraction and XRD (Fig. 4), but a halo pattern with no peaks was observed for any of the specimens. Thus, the resulting Nd-based particles were amorphous. In previous studies, we synthesized Sm-, Pr-, and Dy-doped composite films using urethane resins and evaluated their PL properties.^10–12^ In these studies, Sm, Pr, and Dy ions moved through the urethane resin matrix upon UV irradiation, resulting in the growth of Sm_2_O_3_, Pr_6_O_11_ and Dy_2_O_3_ particles.^10–12^ The size of Nd-based compound particles increase from 15.0 nm to 26.2 nm with increasing UV irradiation time. In PL and laser materials, the particle size has been reported to be one of the factors affecting the fluorescence properties.^14–17^ In this study, the particle size at which the Nd-based nanoparticles exhibited the strongest PL intensity was assumed to be approximately 20 nm. However, further UV irradiation caused an increase in the particle size and the PL intensity of the film significantly decreased. The decrease in the PL intensity causes an increase in the particle size, which is attributed to the efficiency of the excitation light irradiation. This was because the larger particle size reduced the surface area of the particles under excitation light irradiation.

**Fig. 5:**
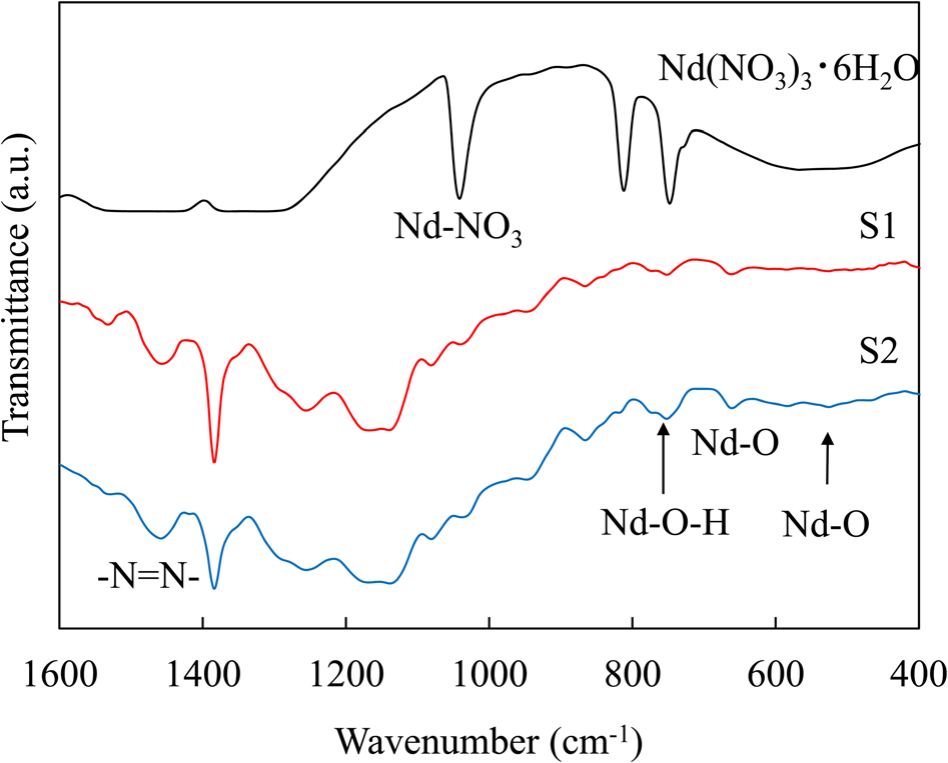
IR spectra of Nd(NO_3_)_3_·6H_2_O and Nd composite films.

Fig. 5 demonstrates the IR spectra of the source Nd (NO_3_)_3_·6H_2_O and Nd composite films (S1, S2). The resulting Nd composite films show a halo pattern corresponding to an amorphous structure by XRD; therefore, we employ FT-IR to identify the chemical species of the particles in the films. The absorption peaks are observed at 522, 653, 730–746, 811, 1041 and 1382 cm^−1^. The peaks at 522 and 653 cm^−1^ are attributed to the characteristic Nd–O vibration originating from Nd_2_O_3_.^18–20^ The peak at 811 cm^−1^ is attributed to nitrate absorption and is estimated to be neodymium nitrate derived from the raw material contained in the matrix.^21,22^ The Nd–O and Nd–O–H stretching vibrations at 730, 746^a^,^22,23^ and 1041 cm^−1^ are attributed to Nd–O stretching from neodymium nitrate hexahydrate.^20^ Neodymium nitrate as the raw material show absorption peaks of Nd nitrate and Nd–OH, whereas the Nd composite films show weak absorption peaks of Nd nitrate and Nd–OH and strong absorption peaks of Nd–O. From the IR spectra of the source neodymium nitrate and the composite films, the source Nd nitrate compound in the matrix decomposes and recombines to form Nd oxide-based compounds (Nd_2_O_3_) upon UV irradiation. Here, similar to the results of TEM observations and our previous studies,^10–13^ it was considered that UV irradiation caused migration of Nd-based compounds in the urethane matrix, resulting in the formation of Nd_2_O_3_ compounds with grain growth. Furthermore, the intensity of the nitrate spectrum decreases and that of the neodymium oxide spectrum increases with increasing UV irradiation time. Neodymium nitrate does not exhibit PL properties. Thus, we confirm that the Nd oxide nanoparticles in the composite films exhibit strong PL properties at a wavelength of 1.06 μm. In the sample immediately after the as cured composite (S1) and the composite with UV light irradiation for 2 hours (S2), N=N bonding was observed at the wavenumber of 1457 cm^−1^,^24,25^ which is a reaction between NH groups in urethane and nitrate groups derived from Nd(NO_3_)_3_, the raw material. The absorption peak at 1457 cm^−1^ due to the N=N bond became larger with increasing the UV irradiation time. These results suggested that the NO_3_ ions reacted with the urethane in the matrix by UV irradiation, forming azo groups and degrading the urethane in the matrix.

## 4. Conclusion

Nd-based nanoparticle-embedded urethane resin composite films were fabricated using neodymium nitrate hexahydrate and urethane resin. The obtained films were light blue, which turned brown because of the reaction of nitrate ions and the urethane matrix under UV irradiation. The composite films exhibited near-infrared emission at 1.06 μm (^4^F_3/2_ → ^4^I_11/2_), originating from Nd^3+^ of neodymium-oxide-based nanoparticles during the irradiation of a halogen lamp. We found that the appropriate UV irradiation conditions for obtaining NIR luminescence were 0–2 h because of the coloration caused by the nitrate ions contained in the film. The particle size of the Nd_2_O_3_ nanoparticles in the prepared Nd composite films ranged from 15.0 to 26.2 nm, and the particle size and dispersion in the composite films were controlled by the UV irradiation time.

In this study, the fabricated Nd composite films exhibited green PL at an NIR PL wavelength of 1.06 μm. We will continue to study the possibility of laser oscillation of Nd-based composite films by pumping them with Xe lamps. Furthermore, prolonged UV irradiation accelerated the degradation of the film because of the reaction between the nitrate ions and urethane resin in the composite film. Therefore, it is necessary to replace the raw materials, *i.e.*, Nd nitrate or urethane matrices.

## Data availability

The data that support the findings of this study are available from the corresponding author, Hidetoshi Miyazaki, upon reasonable request.

## Conflicts of interest

The authors declare no conflict of interest.
